# Silver SERS Adenine Sensors with a Very Low Detection Limit

**DOI:** 10.3390/bios10050053

**Published:** 2020-05-15

**Authors:** Yonhua Tzeng, Bo-Yi Lin

**Affiliations:** Department of Electrical Engineering, National Cheng Kung University, Tainan 701, Taiwan; 03132026@gm.scu.edu.tw

**Keywords:** SERS, silver, adenine, R6G, graphene, plasmon, Raman scattering, copper

## Abstract

The detection of adenine molecules at very low concentrations is important for biological and medical research and applications. This paper reports a silver-based surface-enhanced Raman scattering (SERS) sensor with a very low detection limit for adenine molecules. Clusters of closely packed silver nanoparticles on surfaces of discrete ball-like copper bumps partially covered with graphene are deposited by immersion in silver nitrate. These clusters of silver nanoparticles exhibit abundant nanogaps between nanoparticles, where plasmonic coupling induces very high local electromagnetic fields. Silver nanoparticles growing perpendicularly on ball-like copper bumps exhibit surfaces of large curvature, where electromagnetic field enhancement is high. Between discrete ball-like copper bumps, the local electromagnetic field is low. Silver is not deposited on the low-field surface area. Adenine molecules interact with silver by both electrostatic and functional groups and exhibit low surface diffusivity on silver surface. Adenine molecules are less likely to adsorb on low-field sensor surface without silver. Therefore, adenine molecules have a high probability of adsorbing on silver surface of high local electric fields and contribute to the measured Raman scattering signal strength. We demonstrated SERS sensors made of clusters of silver nanoparticles deposited on discrete ball-like copper bumps with very a low detection limit for detecting adenine water solution of a concentration as low as 10^−11^ M.

## 1. Introduction

**Figure 1:**
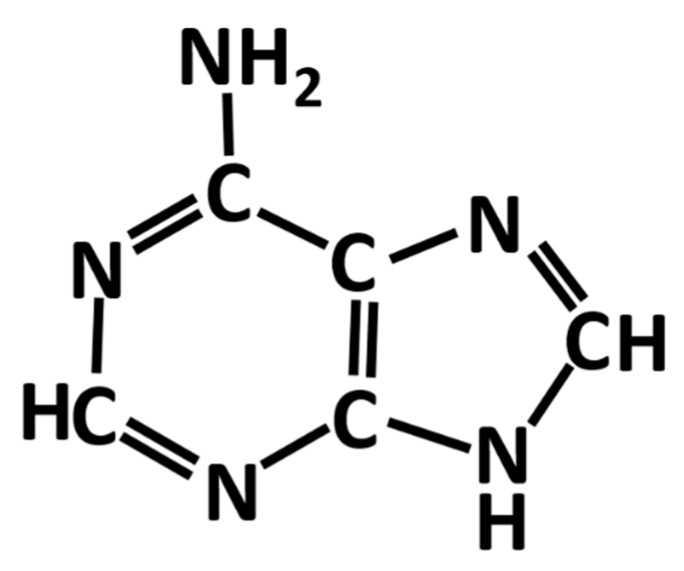
Chemical structure of an adenine molecule.

Adenine is one of aromatic bases found in DNA and RNA. It is soluble in water only at a low concentration. Shown in Figure 1 is a schematic diagram of the chemical structure of an adenine molecule. Adenine adsorption on silver and gold nanoparticles and electrodes exhibits desirable surface-enhanced Raman scattering (SERS) signal strength, which is suitable for the detection of diseases, DNA hybridization and many biomedical and agricultural applications [1]. Therefore, it has become one of the most frequently studied biomolecules along with the rapid increase in SERS research in recent years [2,3,4].

Adenine molecules adsorb on metal surfaces by strong and complex interactions. The interactions include electrostatic forces and coupling by functional groups. In most reported cases, adenine molecules adsorb on silver metal at a tilted angle to the surface [2]. Some planar adsorption was also reported [5]. The strong interactions with silver metal surface lead to the low diffusivity of adenine molecules on silver surface. The characteristics result in different SERS detection limits for adenine molecules from that for Rhodamine 6G molecules [5,6,7,8,9,10,11]. Recently, we reported a novel means of applying nanoscale graphene islands on copper foils as a template or a mask to selectively chemically plate silver nanoparticles on the surface of a copper foil without graphene coverage [12,13]. A two-dimensional array of discrete but closely spaced silver nanoparticles was deposited on a planar copper foil using a graphene template. By gradually increasing the silver deposition time and repetitively measuring the distance between silver nanoparticles, high-density nanoscale silver gaps were optimized. Graphene islands, which serve as a mask to block silver deposition by chemical plating, remain on the copper surface and are surrounded by silver nanoparticles. A low SERS detection limit of R6G molecules well below 10^−12^ M was achieved routinely. By optimizing the gap spacing and the density of nanoscale gaps, 10^−16^ M R6G molecules was detected. This extremely low detection limit by a SERS sensor was found to be not applicable to adenine molecules. The detection limit of adenine molecules by the same SERS sensor was found to be many orders of magnitude higher than that of R6G molecules. For example, a detection limit on the order of 10^−6^ M adenine molecules was measured in comparison with that of 10^−12^–10^−16^ M for R6G molecules. Aiming at finding an effective means of improving the detection limit for adenine molecules, this study was carried out to resolve the difference in sensitivity of SERS sensors to R6G molecules from adenine molecules.

For a SERS sensor to detect a specific kind of molecules at a very low concentration, it is necessary to increase the probability of the molecules to adsorb on sensor, where local electric fields are very high so that the enhanced Raman scattering signal strength is high. Plasmonic coupling has been found to be an effective means of inducing very high local electric fields [14,15]. If a molecule adsorbs in a low-field area, the molecule contributes little to the measured Raman scattering signal strength. The low detection limit that is being pursued is adversely affected. On the other hand, it is necessary for the sensor to have as many as possible “hot spots” where plasmon-induced local electric fields are very high. The main mechanisms for molecules to adsorb on a SERS sensor vary for different molecules. For achieving a low detection limit for different molecules, different SERS design strategies are therefore required. The best SERS sensor for adenine molecules may be different from that for R6G molecules.

The adsorption of molecules on a sensor surface where the local electromagnetic field is weak should be minimized. Instead, adsorption of molecules to “hot spots” where high local electric fields are induced by plasmonic coupling should be promoted. For molecules which interact with and adsorb on silver easily, there should be no silver in the low-field areas. By these means, most molecules will adsorb on a high-field sensor surface and contribute to the measured Raman scattering signal strength. As few as possible molecules are wasted due to adsorption in low-field areas of the sensor.

The detection limit of adenine molecules by gold and silver-based SERS sensors has been reported to range mainly from 10^−8^ M to 10^−9^ M [16,17,18,19,20]. Some research groups reported the detection of adenine molecules at a lower concentration of 10^−10^ M [21] and 10^−11^ M [22]. In order to achieve a 10^−11^ M detection limit, semiconductor nanofabrication technology was applied [22].

R6G molecules, instead of interacting favorably with silver surfaces of positive potential, interact with graphene with a negative potential. When a graphene mask is present below nanogaps formed between silver nanoparticles, R6G molecules are more likely to diffuse on the surface of silver nanoparticles to enter nanogaps where the SERS enhancement factor is the strongest. R6G molecules accumulate in the nanogaps, where the local electromagnetic field induced by plasmonic coupling is very strong and the included Raman scattering signal strength is significantly enhanced. R6G molecules are more likely than adenine molecule to adsorb inside abundant silver nanogaps in an array of close spaced silver nanoparticles deposited on a planar copper surface. This is believed to have resulted in much lower detection limit for R6G molecules than adenine molecules.

## 2. Materials and Methods

**Figure 2:**
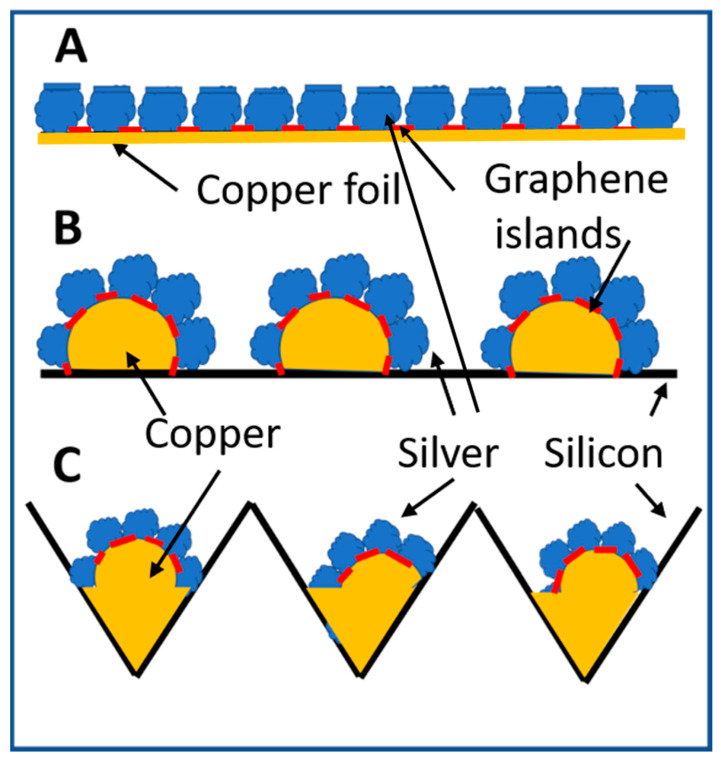
Schematic diagram of silver nanoparticles grown by nitrate reduction on graphene nano-islands template covered copper surfaces. (**A**) Two-dimensional array of silver nanoparticles deposited on a planar copper surface using graphene nano-islands synthesized on copper by rapid thermal CV technique as a template. The red sections represent the graphene template. (**B**) Thermal annealing of a copper thin film deposited on a planar silicon wafer breaks apart the copper thin films into discrete pieces, which peel off from the silicon surface, melt or deform into discrete three-dimensional copper structures. Graphene nano-islands deposited on the copper structures serve as a template for silver nanoparticles to grow by nitrate reduction in directions perpendicular to the copper surfaces. (**C**) A copper thin film is deposited on a silicon wafer with etched holes. Broken copper thin films melt and solidify at the bottom and on the sidewall of etched holes. Discrete silver nanoparticles are deposited on copper surfaces using graphene nano-islands synthesized on copper surfaces as a template.

The process of fabricating a two-dimensional array of silver nanoparticles on a planar copper foil as illustrated in Figure 2A has been described in reference [12,13]. Figure 2B,C show the deposition of clusters of silver nanoparticles on discrete copper structures. The copper structure is formed after the copper thin film on the silicon surface melts at high temperature during annealing and the graphene thermal CVD processes. Copper films are deposited by RF magnetron sputtering in Ar gas using 60 W RF power at 13.56 MHz for 15-min pre-sputter cleaning. It is followed by applying RF power of 90 W for sputter coating of copper films of a desired thickness by controlling the sputtering time. Copper films which are thinner than 100 nm peel off easily from the silicon surface under the copper annealing and graphene growth conditions at 900 °C. Broken, peeling off, and distorted pieces from 100 nm thick copper films scatter around a planar silicon surface. This is illustrated by Figure 2B. Distribution of copper pieces on a planar silicon surface is random. They are not suitable for the fabrication of a reproducible SERS sensor.

In order to collect molten copper in pre-arranged location for achieving uniformly distributed copper structures for subsequent chemical plating of silver nanoparticles, holes are chemically etched into a planar silicon wafer [23]. A sputtered copper film of thinner than 100 nm melts, flows and solidifies inside etched holes. Subsequent rapid thermal CVD of graphene on the ball-like copper bumps creates a template for the selective deposition of silver nanoparticles only on copper surfaces where there are no graphene islands to block the chemical plating of silver. Silver grows vertically from ball-like copper bumps surface to form a cluster of elongated nanoparticles pointing in different directions as illustrated by Figure 2C. Table 1 and Table 2 show the remaining detailed process parameters for hole etching, thermal annealing of copper thin films, and rapid thermal CVD of graphene islands on copper.

Silver nitrate solution was prepared by adding 100 mL deionized water to 0.08493 g of silver nitrate powder (Sigma-Aldrich, Inc., purity >99.8%, St. Louis, MO, USA) at room temperature followed by thorough stirring. SERS samples were immersed in 3 mL silver nitrate solution for a pre-determined period of time and then removed for blowing dry by nitrogen. Chemical plating of silver on copper surface, where there is no graphene deposition was applied to deposit silver nanoparticles. In the chemical plating of silver on copper, copper metal dissolves in the silver nitrate solution. Silver deposits on the copper surface [24]. Silver does not deposit on graphene surface. The balanced equation for the reaction is

Cu(s) + 2AgNO_3_(aq)------->Cu(NO_3_)_2_(aq) + 2Ag (1)

According to Product Number A8626, CAS #: 73-24-5, Sigma-Aldrich, Inc., adenine is soluble at 1 part to ~2000 parts cold water. In order to ensure full dissolution, adenine (Sigma, purity >99%) of 0.5 mg was dissolved in deionized water to make 3.7 × 10^−6^ M adenine in water solution. The solubility of adenine in water is low and depends on temperature and other conditions. The initial concentration of adenine in water solution at 3.7 × 10^−6^ M is well below the solubility of adenine in water at room temperature. This adenine solution was further diluted by deionized water by 10 times each time and repetitively to prepare a series of adenine water solution of different concentration. The order of magnitude of the concentrations, i.e., 10^−6^, 10^−7^, 10^−8^, 10^−9^, 10^−10^, 10^−11^, 10^−12^ M were used for evaluation of SERS sensors.

After the SERS sensor was immersed in adenine water solution of a known concentration for 10 min, the sensor was removed from the solution and blown dry by nitrogen. The Horiba Scientific Raman system with a green laser at 532 nm and a laser power at 450 mW was used to measure Raman spectra. The Raman system has an optical fiber optical system with a 100 times object lens. The laser focus was optimized to measure the highest Raman scattering signal of the test sample. Diamond crystals with a Raman peak at 1332 cm^−1^ are often used for calibration. The laser beam was focused on the sensor surface in an area of about 10 μm in size. Raman spectra were measured in 5–10 different areas on each sensor for each concentration of adenine water solution. The concentration of adenine is considered to be above the low detection limit if the Raman scattering peak at 760 cm^−1^, which is characteristic of adenine molecules, is clearly distinguishable from the background signal. The low detection limit was determined by the lowest concentration of adenine solution, which produced a clearly detectable adenine Raman scattering peak. Multiple sensors were characterized.

## 3. Results and Discussion

For the same spacing between neighboring silver particles, the larger the aspect ratio of the silver particles is, the higher the field enhancement is on the top surface of silver particles. For the same aspect ratio of closely spaced silver particles, the larger the spacing is, the higher field enhancement is on the top of silver particles. Therefore, closely spaced silver particles, which are arranged in a two-dimensional array and with an aspect ratio being near one on a planar surface as shown in Figure 2A exhibit weak geometric electromagnetic field enhancement on the top surface of silver particles. The geometric field enhancement is minor compared to the very strong local electric fields induced by plasmonic coupling, though. Unlike nanoscale gaps between silver nanoparticles, the top surfaces of silver nanoparticles do not form “hot spots” with very high local electric fields. Adenine molecules, which adsorb on low-field top surface of silver nanoparticles due to strong interactions with silver contribute little to the measured Raman scattering signal strength. When a very low concentration of adenine molecules in water is available, the more the adenine molecules adsorb on low-field area, the less the remaining adenine molecules are available to adsorb at ‘hot spots’ in silver nano-gaps. This results in the need for a higher concentration of adenine molecules for a SERS sensor to be able to detect and thus, a higher and less desirable detection limit.

Table 2 illustrates the fact that electronegative graphene islands, which serve as a mask to block the chemical plating of silver nanoparticles on graphene covered planar copper foils, remain at the bottom of silver nanogaps. R6G molecules do not interact as strongly as adenine molecules do with silver and can diffuse more freely on silver surface. It is favorable for R6G molecules with positive potential to be trapped inside nano-gaps near graphene on a copper surface. Therefore, even at a very low concentration, R6G molecules can produce a strong enough Raman scattering signal strength to be detected by the Raman system. SERS sensors made of two-dimensional arrays of silver nanoparticles with a high-density of nanogaps on planar sensor surfaces are, therefore, capable of detecting a very low concentration of R6G molecules.

On the other hand, in addition to coupling by functional groups with silver, adenine molecules exhibit a negative potential. Adenine interacts strongly with silver surface of positive potential [1,2]. When an adenine molecule arrives near the top surfaces of a two-dimensional array of silver nanoparticles, it is attracted by the positive potential of silver surface to adsorb on the top surface of a silver nanoparticle where the local electromagnetic field is weak. This adenine molecule does not contribute much to the measured Raman scattering signal strength and is wasted from the viewpoint of pursuing a low detection limit. This is especially a severe situation when the available adenine molecules are of a small number. Therefore, the strategy of minimizing the silver surface area on a SERS sensor where local electromagnetic field is weak is promising for collecting as many adenine molecules as possible in areas of high local electric fields.

Figure 2A illustrates a two-dimensional array of silver nanoparticles deposited by the reduction of silver nitrate on copper surfaces using graphene islands as a mask. Silver deposits on copper surface but not on graphene surface. The deposited silver nanoparticles on a SERS sensor form a very high density of nanoscale silver gaps. However, the top surfaces of the two-dimensional array of silver nanoparticles forms a virtual flat surface, where geometric electromagnetic field enhancement is low.

When a laser beam illuminates the SERS sensor, the electromagnetic wave of the laser beam drives electrons in neighboring silver nanoparticles in the same direction opposite to the local electric fields. The electron drifting results in the re-distribution of electrons in silver nanoparticles. When the electromagnetic field of the laser beam is in a direction across a silver nanogap, high-density charges of opposite signs gather on two counter-surfaces inside a nano-gap, where a strong local electromagnetic field is induced.

Adenine molecules interact with silver strongly and do not diffuse freely on silver surfaces. Closely spaced silver particles with an aspect ratio being close to one on a planar surface, as illustrated by Figure 2A, exhibit little geometric electromagnetic field enhancement. SEM images of the silver nanoparticles are reported in [12,13]. The probability for an adenine molecule to adsorb on the top surface of a silver nanoparticle is higher than inside the nanogaps because nanogaps are very small compared to the remaining surfaces of silver nanoparticles. Under this condition, it is quite a waste of a limited supply of low concentration adenine molecules because many of them do not contribute much to the measured Raman scattering signal strength for effective detection of their presence. It is more desirable for adenine molecules to remain mobile after they arrive at the surfaces of silver nanoparticles so that they have the opportunity to move around and adsorb inside nanogaps. However, the strong interaction between adenine and silver metal suppresses the surface diffusion of adenine molecules. As a result, only a small portion of the adenine molecules contribute to the Raman scattering signal strength. The SERS detection limit for Adenine molecules by SERS sensors with planar arrays of silver nanoparticles, which exhibit more low-field silver surfaces than high-field surfaces is therefore not as low as desired.

**Figure 3:**
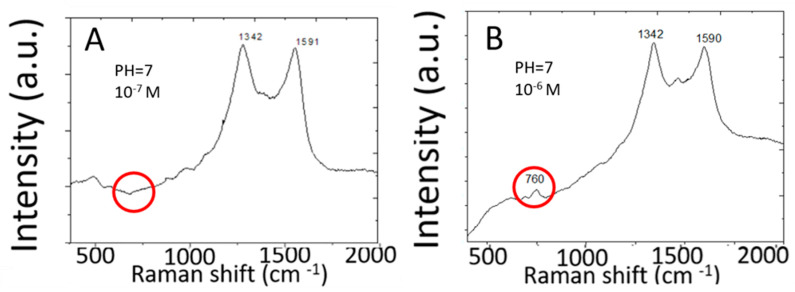
SERS spectra of adenine in water measured by two-dimensional array of silver nanoparticles on a planar copper foil. (**A**) 10^−7^ M, (**B**) 10^−6^ M.

Figure 3 shows Raman spectra measured from 10^−6^ M and 10^−7^ M adenine molecules in water by two-dimensional silver arrays deposited on a planar copper substrate. Raman spectra measured from 10^−7^ M adenine solution do not show Raman peaks characteristic of adenine. The detection limit for adenine is only 10^−6^ M. This detection limit is more than six orders of magnitude higher than the detection limit for R6G by the same SERS sensor made of high-density two-dimensional array of silver nanoparticles on planar copper foils. Discrete graphene islands are deposited by rapid thermal CVD to serve as a template (or a mask) for subsequent silver deposition by chemical plating. Discrete graphene islands exhibit abundant edges characterized by different Raman spectra from that of continuous monolayer graphene. Different Raman peaks in the Raman spectra of discrete graphene islands are further enhanced to different extents by the silver nanoparticles. The Raman spectra of graphene islands on copper are further complicated by the strong background signal from the copper substrate. In order to clearly characterize the graphene islands, graphene islands were transferred to silicon wafers with 300 nm silicon dioxide on the surface. Please refer to [12] for details of Raman spectra and analysis.

**Figure 4:**
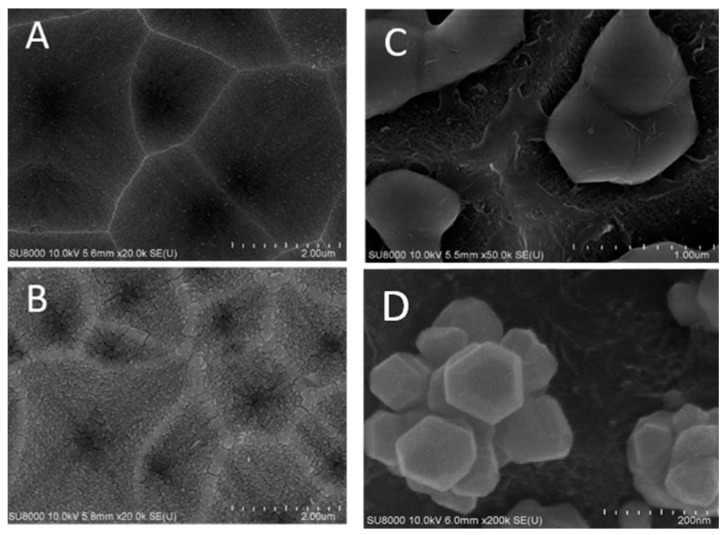
SEM images of (**A**) etched holes in a silicon wafer; (**B**) copper thin films on surfaces of etched holes; (**C**) solidified three-dimensional copper bumps inside holes etched into the silicon wafer; (**D**) clusters of silver nanoparticles grown in varied orientations by silver nitrate reduction on copper surfaces which are partially covered by graphene nano-islands synthesized by rapid thermal CVD.

In order to control the distribution of silver clusters better, holes were etched into silicon substrates before copper thin films were deposited. Figure 4A shows an SEM image of etched holes in a silicon wafer. Figure 4B shows an SEM image of a copper thin film of 75 nm thick on silicon surface with etched holes. RF magnetron sputtering technique is applied for the copper deposition. Figure 4C shows an SEM image of solidified copper pieces from a molten 75 nm thick copper thin film. The copper film breaks, melts, and then solidifies to form ball-like copper bumps inside etched holes on a silicon wafer. The copper annealing and rapid thermal CVD of graphene template cause the 75 nm copper thin film to melt, separate and form ball-like copper bumps, which adhere on silicon surfaces inside etched holes.

Figure 4D shows an SEM image of clusters of silver nanoparticles deposited on exposed copper surfaces not covered by graphene by immersion in silver nitrate solution. These silver clusters are oriented in different directions depending on where the ball-like copper bumps adhere in etched holes. Molten copper structures, which adhere inside holes etched into silicon will appear different depending whether they adhere at the bottom or on sidewalls of an etched hole.

The schematic illustration shown in Figure 2C shows that unlike arrays of silver nanoparticles shown in Figure 2A, discrete silver clusters do not exhibit a planar surface formed by the top surfaces of silver particles. When an adenine molecule adsorbs on the planar silver surface illustrated by Figure 2A, the adenine molecule is not located in an area with a strong local electric field. For SERS detection of adenine molecules at the lowest possible concentration, the efficiency for adenine molecules to adsorb on silver surfaces with high local electric fields is important and required. The discrete clusters of silver nanoparticles grown on ball-like copper bumps are thus promising to provide an adenine SERS sensor with a very low detection limit. The detection limit of adenine molecules improves many orders of magnitude by the clusters of silver nanoparticles grown in varied orientations on ball-like copper bumps structures in comparison with planar silver arrays.

**Figure 5:**
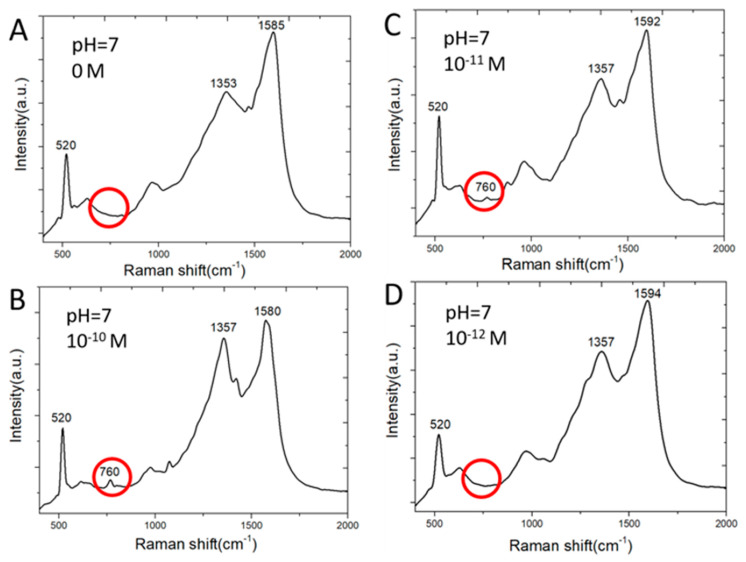
Raman spectra of adenine molecules in water having absorbed on SERS sensor surface: (**A**) 0 M; (**B**) 10^−10^ M; (**C**) 10^−11^ M; (**D**) 10^−12^ M.

Figure 5 shows SERS spectra of adenine molecules. Adenine is detected by one of major Raman scattering peaks at 760 cm^−1^. Clusters of silver nanoparticles grown on ball-like copper bumps and masked by graphene as shown in Figure 4D and Figure 2C achieve a much lower detection limit than the two-dimensional array of silver nanoparticles on planar copper surface [12,13] shown in Figure 2A,B. This is among the lowest reported detection limits of adenine molecules by silver-based SERS sensors [21]. On the contrary, copper deposited on etched holes in a silicon wafer without using a graphene mask results in the deposition of larger silver particles scattering around wherever copper is present both in etched holes and on the flat silicon surface. These silver nanoparticles are not effective in producing strong Raman scattering signal strength. The silver SERS sensor made without graphene masks on nonplanar copper does not detect adenine of concentration lower than 10^−6^ M. On the other hand, silver SERS sensors made with graphene nano-islands on planar copper cannot detect adenine of a concentration below 10^−6^ M. However, it does allow the optimization of silver nanogaps as reported in [12,13] for detecting R6G molecules of a concentration as low as 10^−16^ M. Graphene islands-based template plays important roles on where silver particles can be deposited on copper surface. The etched holes allow much thinner copper to be deposited, melted, and collected as non-planar copper bumps inside the etched holes. The combined effects of the etched holes and the graphene template result in favorable clusters of silver nanoparticles for achieving a very low detection limit of adenine in water.

Table 3 shows the detectable range of adenine concentration (marked by o) by SERS sensors with silver nanoparticles grown on ball-like copper bumps. By adjusting the silver deposition time, both the density of silver nanoscale gaps and the large-curvature silver surfaces are maximized. In areas between clusters of silver nanoparticles, the local electromagnetic field is low and no silver is deposited.

**Figure 6:**
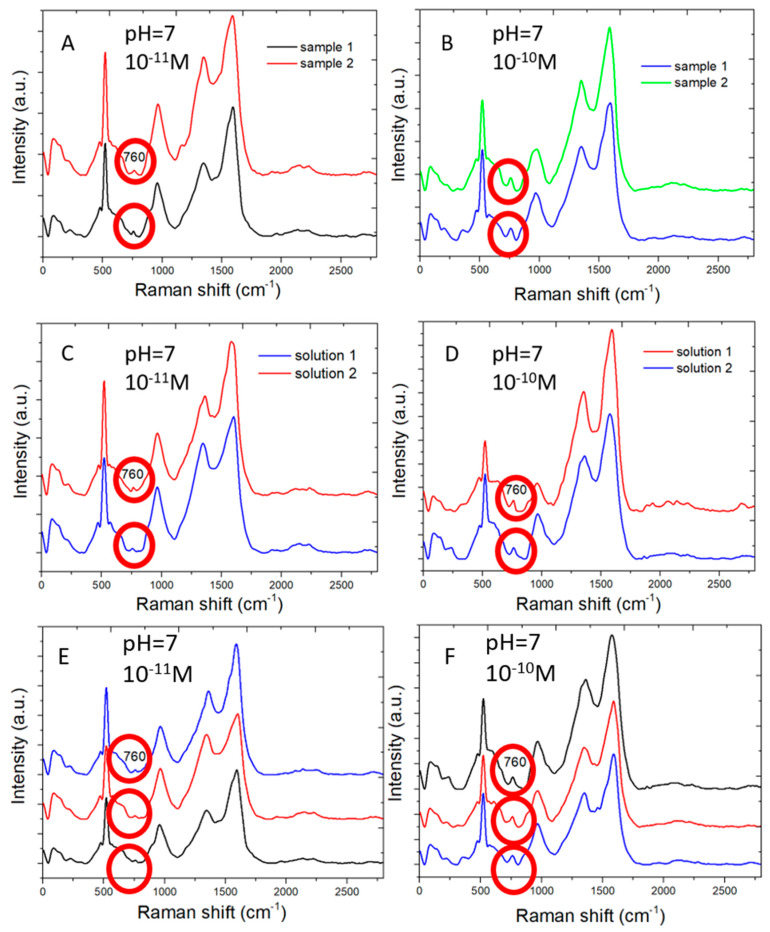
Multiple Raman spectral measurements: (**A**) Raman spectra measured from 10^−11^ M adenine molecules in water by two different SERS sensors. (**B**) The same as (**A**) but for 10^−10^ M concentration. (**C**) Raman spectra measured from two different water solution of 10^−11^ M adenine by the same SERS sensor. (**D**) The same as (**C**) but for two water solution of 10^−10^ M adenine. (**E**) Raman spectra measured at three different areas of a SERS sensor from 10^−11^ M adenine molecules in water. (**F**) The same as (**E**) but for 10^−11^ M adenine molecules in water.

The SERS sensors measure adenine molecules in water solution in good reproducibility. Figure 6 shows Raman spectral measured by two different SERS sensors from adenine molecules of concentration 10^−11^ M (Figure 6A) and 10^−10^ M (Figure 6B) in water, respectively. Similar Raman peaks are shown for each of two low concentration of adenine. This shows the reproducibility of SERS sensors.

Figure 6C,D shows the Raman spectra measured by the same SERS sensors from two sets of adenine water solution of 10^−11^ M (Figure 6C) and 10^−10^ M (Figure 6D). Each set includes two water solutions, which are separately prepared. The SERS sensor clearly distinguishes adenine molecules of two different concentrations and shows similar Raman signal intensity for adenine of the same concentration.

Figure 6E,F shows the Raman spectra measured in different areas on the same SERS sensor for adenine molecules of 10^−11^ M (Figure 6E) and 10^−10^ M (Figure 6F) in concentration. Similar Raman spectra are measured for the same concentration in different areas of a sensor. The variation in Raman scattering signal intensity measured in different areas is expected for low concentration of adenine molecules near the low detection limit because the number of adenine molecules adsorbing on the sensor surface is small. The distribution of those adenine molecules adsorbing on the sensor surface is not perfectly uniform.

**Figure 7:**
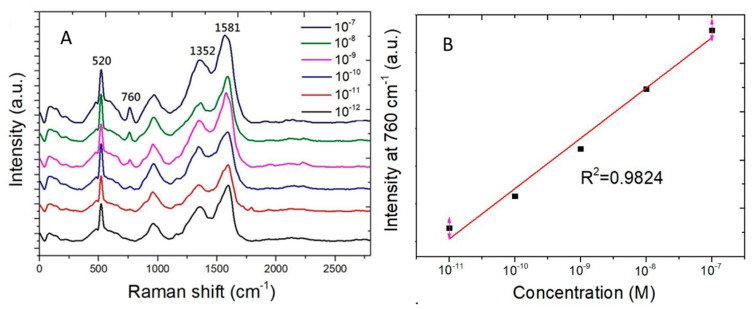
Raman scattering peak at 760 cm^−1^ measured from adenine water solution of the concentration ranging from 10^−7^ M to 10^−12^ M. (**A**) Raman spectra. (**B**) The Raman scattering signal strength at 760 cm^−1^ as a function of adenine concentration in water. The curve fitting shows a coefficient of determination (R^2^) being 0.9824.

Figure 7A shows the Raman spectra measured from adenine water solution of the concentration ranging from 10^−7^ M to 10^−12^ M. The Raman scattering signal strength at 760 cm^−1^ decreases with concentration of adenine molecules in water. Figure 7B shows the Raman scattering signal strength at 760 cm^−1^ as a function of the adenine concentration. The vertical axis is of linear scale. The horizontal axis is of logarithmic scale of the adenine concentration. A linear curve fitting of experimental data shows a coefficient of determination (R^2^) being 0.9824. The coefficient of determination being nearly one shows that the good preciseness of the linear curve fitting for the Raman signal strength measured from adenine molecules which adsorb on SERS sensors.

The interaction of molecules with silver surface is complex. For an adenine SERS sensor of very low detection limit, it is desired for the density of high-field areas, i.e., “hot spots”, to be large and for silver surface with low local electric fields to be small. The combined effect is for the sensor to allow most of molecules on sensor surface to be in high-field areas in order to make significant contribution to the measured Raman scattering signal strength. Both electrostatic force and coupling by functional groups with silver affect adversely the diffusivity of adenine molecules on silver surface. Therefore, for as much as possible silver surface on an adenine SERS sensor to be in high-field area is especially important. Clusters of closely packed silver nanoparticles chemically plated selectively on ball-like copper bumps surface without coverage by the graphene mask help satisfy the requirement. Silver nanoparticles grown perpendicularly on ball-like copper bumps create abundant silver nano-gaps, where plasmonic “hot spots” are present. There is no copper on the rest of the sensor surface surrounding ball-like copper bumps. No field enhancement in these areas, where no silver is deposited. Therefore, adenine molecules are less likely to adsorb on sensor surface of low local electric fields. Most adenine molecules, which adsorb on the sensor will absorb on silver surface on ball-like copper bumps and contribute to the measured Raman scattering signal strength by the aid of plasmonic coupling at nanogaps between silver nanoparticles. Therefore, adenine molecules in a low concentration water solution down to 10^−11^ M can be detected.

A similar process (not shown) was carried out in optimizing the thickness of deposited copper thin film and time for etching holes in silicon wafers. A copper thin film of 75 nm was applied for demonstrating the very low detection limit of 10^−11^ M adenine in water. Our preliminary results show that the very low detection limit of the reported SERS adenine sensor decreased further by at least one order of magnitude by adjusting the pH level of the water solution. Interactions of adenine molecules in water with silver depend on the pH level of the water solution. Details of the effects of the pH level on the very low detection limit of adenine molecules in water by SERS sensors will be published elsewhere in the near future.

The intended purpose of this analysis was to prove that the novel SERS design strategy solves the problem with poor low detection limit of adenine molecules in water by our previously demonstrated excellent SERS sensor for detecting R6G molecules. This analysis adequately serves the intended purpose. Low detection limit is only one of several essential issues of SERS sensors. Our prior publications demonstrated SERS sensors of excellent reproducibility, re-usability, durability, environmental compatibility and shelf lifetime. Our prior publication [8] has reported excellent SERS sensors, which are re-usable, precise, and of high reliability and environmental compatibility. How to invent a SERS sensor with all the excellent and desirable characteristics is certainly in our mind. It is hopeful that this wish will be accomplished in our future research work.

## 4. Conclusions

A very low detection limit of 10^−11^ M adenine molecules in water was achieved. SERS sensors based on clusters of silver nanoparticles grown by silver nitrate reduction on discrete three-dimensional copper bumps, which are partially covered by graphene nano-islands. Because the strong interactions of adenine molecules with positively charged silver surfaces, adenine molecules adsorb on both silver surfaces with high and low local electric fields. By reducing the planar surface areas, a silver SERS sensor utilizes the limited supply of adenine molecules from a very low concentration of adenine solution in water much more efficiently. Clusters of silver nanoparticles on non-planar copper surfaces exhibit surfaces of high curvature and high-density nanogaps. Moreover, the low-field areas between ball-like copper bumps do not have silver deposition. Adenine molecules are less likely to adsorb on low-field areas. Adenine molecules adsorb on these high-field areas generate strong SERS signal strength to enable the detection of very low concentration of adenine molecules.

## Figures and Tables

**Table 1:** Process Parameters for Etching Holes in Silicon Wafers.

Step 1	Step 2	Step 3
Etching SiO_2_ layer	Etching Si layer	Clean with DI water
H_2_O_2_ 10 mL HF 10 mL	CuSO_4_ 40 mM H_2_O_2_ 10 mL H_2_O 50 mL HF 13 mL	DI water
Immersion 1 h	etching 30 min	

**Table 2:** Process Parameters for Copper Annealing and Graphene Rapid Thermal CVD.

Step 1	Step 2	Step 3	Step 4
Silicon etching & Suptter deposition of copper thin film	900 °C copper annealing 30 s	900 °C growth graphene nano island 40 s	Immersion in AgNO_3_
	H_2_: 15 sccm	H_2_: 15 sccm CH_4_: 20 sccm	
	0.3 Torr	0.5 Torr	

**Table 3:** Low Detection Limit of Adenine Using SERS Sensors Made of Silver Nanoparticles Deposited for Different Length of Time on Ball-like Copper Bumps Formed from 75-nm-thick Copper Films.

Adenine (M)	Silver Deposition Time						
	1 min 15 s	1 min 30 s	1 min 45 s	2 min	2 min 15 s	2 min 30 s	2 min 45 s
10^−12^							
10^−11^			o	o			
10^−10^		o	o	o	o	o	
10^−9^	o	o	o	o	o	o	o

## References

[1] Harroun S.G. (2018). The Controlversial Orientation of Adenine on Gold and Silver. ChemPhysChem.

[2] Giese B., McNaughton N. (2002). Surface-Enhanced Raman Spectroscopic and Density Functional Theory Study of Adenine Adsorption to Silver Surfaces. J. Phys. Chem. B.

[3] Lin H., Mock J., Smith D., Gao T., Sailor M. (2004). Surface-Enhanced Raman Scattering from Silver-Plated Porous Silicon. J. Phys. Chem. B.

[4] Girel K., Yantcevich E., Arzumanyan G., Doroshkevich N., Bandarenka H. (2016). Detection of DNA molecules by SERS spectroscopy with silvered porous silicon as an active substrate. Phys. Status Solidi A.

[5] Xiao Y.-J., Chen Y.-F., Gao X.-X. (1999). Comparative study of the surface enhanced near infrared Raman spectra of adenine and NAD+ on a gold electrode. Spectrochim. Acta Part A Mol. Biomol. Spectrosc..

[6] Mirajkar S., Dhayagude A., Maiti N., Suprasanna P., Kapoor S. (2019). Distinguishing genomic DNA of Brassica juncea and Arabidopsis thaliana using surface-enhanced Raman scattering. J. Raman Spectrosc..

[7] Kimura-Suda H., Petrovykh D.Y., Tarlov M.J., Whitman L. (2003). Base-Dependent Competitive Adsorption of Single-Stranded DNA on Gold. J. Am. Chem. Soc..

[8] Chen S.-T., Chu Y.-C., Liu C.-Y., Huang C.-H., Tzeng Y. (2012). Surface-enhanced Raman spectroscopy for characterization of nanodiamond seeded substrates and ultrananocrystalline diamond at the early-stage of plasma CVD growth process. Diam. Relat. Mater..

[9] Liu C.-Y., Liang K.-C., Chen W., Tu C.-H., Liu C.-P., Tzeng Y. (2011). Plasmonic coupling of silver nanoparticles covered by hydrogen-terminated graphene for surface-enhanced Raman spectroscopy. Opt. Express.

[10] Huang C.-H., Lin H.-Y., Chen S., Liu C.-Y., Chui H.-C., Tzeng Y. (2011). Electrochemically fabricated self-aligned 2-D silver/alumina arrays as reliable SERS sensors. Opt. Express.

[11] Huang C.-H., Lin H.-Y., Lau B.-C., Liu C.-Y., Chui H.-C., Tzeng Y. (2010). Plasmon-induced optical switching of electrical conductivity in porous anodic aluminum oxide films encapsulated with silver nanoparticle arrays. Opt. Express.

[12] Tzeng Y., Chen Y., Lai J., Huang B. (2020). Silver Nanoparticles SERS Sensors Using Rapid Thermal CVD Nanoscale Graphene Islands as Templates. IEEE Trans. Nanotechnol..

[13] Tzeng Y., Chen Y.-R. (2019). Carrier for Raman Spectroscopy and Method of Manufacturing the Same. U.S. Patent.

[14] Jain P.K., El-Sayed M.A. (2010). Plasmonic coupling in noble metal nanostructures. Chem. Phys. Lett..

[15] Anderson W.J., Nowinska K., Hutter T., Mahajan S., Fischlechner M. (2018). Tuning plasmons layer-by-layer for quantitative colloidal sensing with surface-enhanced Raman spectroscopy. Nanoscale.

[16] Wang Y.-Y., Cheng H.-W., Chang K.-W., Shiue J., Wang J.-K., Wang Y.-L., Huang N.-T. (2019). A particle-based microfluidic molecular separation integrating surface-enhanced Raman scattering sensing for purine derivatives analysis. Microfluid. Nanofluidics.

[17] Villa J.E., Afonso M.A., Dos Santos D.P., Mercadal P.A., Coronado E.A., Poppi R.J. (2019). Colloidal gold clusters formation and chemometrics for direct SERS determination of bioanalytes in complex media. Spectrochim. Acta Part A Mol. Biomol. Spectrosc..

[18] Lee Y.-C., Chiu C.-W. (2019). Immobilization and 3D Hot-Junction Formation of Gold Nanoparticles on Two-Dimensional Silicate Nanoplatelets as Substrates for High-Efficiency Surface-Enhanced Raman Scattering Detection. Nanomaterials.

[19] Chen G., Zhang K., Luo B., Hong W., Chen J., Chen X. (2019). Plasmonic-3D photonic crystals microchip for surface enhanced Raman spectroscopy. Biosens. Bioelectron..

[20] Zhou N., Meng G., Zhu C., Chen B., Zhou Q., Ke Y., Huo D. (2018). A silver-grafted sponge as an effective surface-enhanced Raman scattering substrate. Sens. Actuators B Chem..

[21] Juang R.-S., Wang K.-S., Cheng Y.-W., Fu C.-C., Chen W.-T., Liu C.-M., Chien C.-C., Jeng R.-J., Chen C.-C., Liu T.-Y. (2019). Floating SERS substrates of silver nanoparticles-graphene based nanosheets for rapid detection of biomolecules and clinical uremic toxins. Colloids Surf. A Physicochem. Eng. Asp..

[22] Ikegami K., Sugano K., Isono Y. Surface-enhanced Raman spectroscopy analysis of DNA bases using arrayed and single dimer of gold nanoparticle. Proceedings of the IEEE MEMS 2017.

[23] Gondek C., Lippold M., Röver I., Bohmhammel K., Kroke E. (2014). Etching Silicon with HF-H2O2-Based Mixtures: Reactivity Studies and Surface Investigations. J. Phys. Chem. C.

[24] Yang T., Ren S., Zhao H. (2019). Introduction of copper ions to regulate silver nanostructures by galvanic displacement reaction. Mater. Res. Express.

